# Brodie’s Abscess of Posterior Ilium with Gluteal Syndrome, an Unusual Cause of Paediatric Low Back Pain: A Case Report

**DOI:** 10.5704/MOJ.1707.009

**Published:** 2017-07

**Authors:** G Behera, M Poduval, DK Patro, S Sahoo

**Affiliations:** Department of Orthopaedics, Jawaharlal Institute of Postgraduate Medical Education and Research (JIPMER), Puducherry, India

**Keywords:** low back pain, Brodie’s abscess, posterior ilium, gluteal syndrome

## Abstract

Brodie’s abscess is a variety of subacute osteomyelitis with a long duration of presentation and intermittent pain. It usually involves the metaphyseal region of long bones of the lower limbs. Brodie’s abscess of pelvic bone is very rare. Involvement of posterior ilium with gluteal syndrome is extremely unusual and can be easily missed or misdiagnosed. We present a 9-year old boy who reported to us with intermittent low back pain of three months duration without any other constitutional symptoms. Clinically, there was mild tenderness over the posterior ilium. Computed tomography showed a lytic lesion in the posterior ilium with a breach in the outer cortex. MRI and bone scan were suggestive of inflammatory pathology. Keeping infective, tubercular and benign bone tumors as differential diagnoses, open biopsy and curettage were done. Staphylococcus aureus was cultured and histopathology was suggestive of osteomyelitis. The patient received appropriate antibiotics for six weeks. He was asymptomatic till 18 months of follow up without any recurrence. We present this case because of its rarity and unusual presentation as gluteal syndrome and low back pain, and its resemblance to other pelvic and sacroiliac joint pathologies which are often missed or misdiagnosed in paediatric patients.

## Introduction

Brodie’s abscess is a type of subacute osteomyelitis first described by Sir Benjamin Brodie in 1832 involving the metaphyseal region of long bones of the lower limb. Pelvic osteomyelitis constitutes only 2 to 3% in children and adolescents, and the ilium is most commonly affected^1^. It may present as lumbar, gluteal and abdominal syndromes depending upon the direction of spread of the inflammation. Spread through the outer cortex into the gluteal muscles presents with pain and swelling in the gluteal region and called gluteal syndrome. We describe here the clinical, radiological and diagnostic features, the management and prognosis with a brief review of the literature.

## Case Report

A nine-year old male student presented with low back pain of three months duration. The pain was intermittent in nature with no history of fever or other constitutional symptoms. There was no history of night pain or contact with tuberculosis. On physical examination, there was a vaguely palpable tender swelling over the right posterior iliac crest just superolateral to the sacroiliac joint. There was no local warmth over the swelling. Clinically, pelvic compression was negative, and the sacroiliac joint appeared normal. There was no history of any intramuscular gluteal injection in the recent past.

**Fig. 1:**
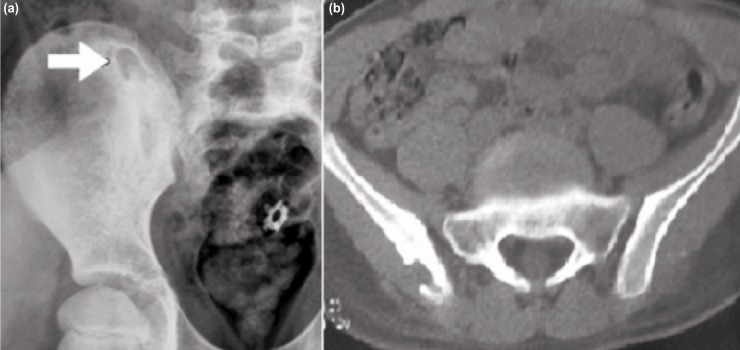
(a) Radiograph of a lytic lesion with surrounding sclerosis in the right posterior ilium and (b) CT scan showing a breach in the outer cortex of the ilium.

**Fig. 2:**
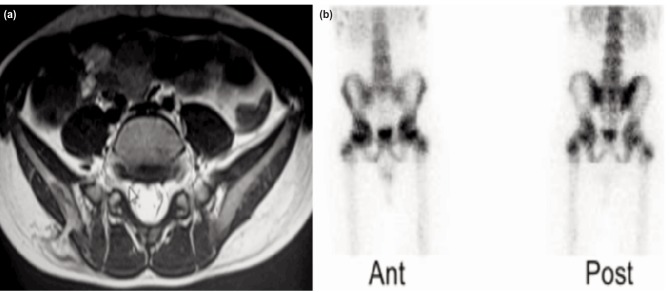
MRI-2 hyper-intense foci; intramedullary and in subcutaneous plane with enhancement 99mTc-MDP scan increased tracer uptake in the right posterior ilium.

Routine blood investigations revealed haemoglobin of 12.6 g/dl, total leucocyte count 10000/mm3, neutrophils 80/100, ESR 31mm at one hour, negative C-reactive protein, serum ADA (adenosine deaminase) 29 IU/L and a negative Mantoux test (2mm). Plain radiograph of the pelvis showed a well-defined lytic lesion with surrounding osteosclerosis in the right posterior iliac crest just superolateral to the ipsilateral sacroiliac joint. CT scan revealed a pure lytic lesion (15 x 10 x 7.5mm) with a breach in the outer cortex of ilium with surrounding sclerosis (Fig. 1). MRI of pelvis showed two hyper-intense foci (T2 & STIR), one intramedullary and one in the subcutaneous plane with diffuse enhancement on contrast suggestive of phlegmonous foci (Fig. 2). Both sacroiliac joints and hip joints were normal. Bone scan with 99mTc-MDP was suggestive of inflammatory/infective pathology involving the right posterior iliac cortex. Fine needle aspiration cytology of the swelling was non-conclusive. Differentials considered were subacute osteomyelitis, tuberculosis, benign bone tumors and eosinophilic granuloma.

**Fig. 3:**
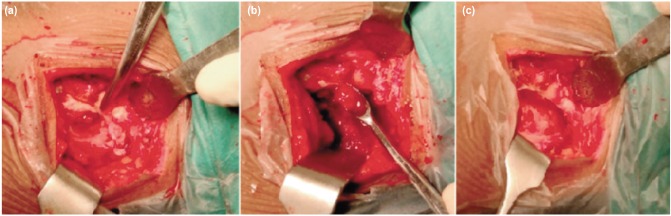
Intra-operative photograph showing (a) A breach in the outer cortex with unhealthy granulation tissue, (b) Curettage and (c) Post-curettage status.

The patient was planned for open biopsy and curettage under general anaesthesia. With the patient in prone position, incision was made over the vague swelling on the right posterior iliac crest. A breach in the outer cortex was noticed with an intramedullary cavitary lesion filled with unhealthy granulation tissue extending into the subcutaneous plane (Fig. 3). The lesion was thoroughly curetted. After through debridement, the wound was closed over a suction drain. The curetted material was submitted for culture and sensitivity tests, acid-fast bacillus stain and histopathological study. Culture of the material revealed growth of *Staphylococcus aureus* sensitive to oxacillin, gentamycin, and vancomycin. Histopathology was suggestive of osteomyelitis. The child received cloxacillin 500mg qid for a total period of six weeks (intravenous for 2 weeks and oral for 4 weeks). He was followed up for 18 months, and no recurrence was noted.

## Discussion

Brodie’s abscess is one variety of subacute osteomyelitis which involves the metaphysis of long bones with indolent clinical features. Pelvic osteomyelitis with gluteal syndrome is very rare in healthy children^1^. The ilium is more commonly affected most probably because of its larger area and rich vascularity.

According to Beaupre and Carroll^2^, pelvic osteomyelitis can present as three different clinical syndromes, namely *lumbar*, *gluteal* or *abdominal* syndromes depending on the spread of inflammation. In the *lumbar type*, the lesion breaches the inner cortex of ilium to the true pelvis and irritates the lumbar plexus and manifests as low backache, hip pain, thigh pain and difficulty in walking. When the inflammation enters the gluteal muscles through a breach in the outer cortex, it presents as *gluteal syndrome*. The most common complaint is buttock pain. Clinically there is local tenderness with or without swelling, and pelvic compression is usually positive. Very rarely, a sinus may be present. In the *abdominal syndrome*, it spreads into the iliac fossa and may present as an acute abdomen (acute appendicitis) or as chronic abdominal pain. Out of five cases of pelvic osteomyelitis in children reported by Beslikas *et al*^1^, two of them had gluteal syndromes, one abdominal syndrome, one presented with lumbar pain and one had features of both gluteal and abdominal syndromes. Similarly, in a study carried out by Rand *et al*^3^ two of four cases had gluteal syndromes while the other two presented as hip pain.

Diagnosing pelvic osteomyelitis is not easy because of its rarity, indolent clinical course, lack of early radiographic signs and absent or borderline haematological parameters^4^. Plain radiograph of Brodie’s abscess shows lytic lesion with peripheral osteosclerosis. Balaji *et al*^5^ in a similar type of case recommended a routine oblique view radiograph of the pelvis in these cases. But in the early stage, radiograph as well as computed tomography are negative. A bone scan is helpful in early diagnosis. MRI has been shown to be very sensitive and in some cases specific for diagnosis. The characteristic pattern includes an outer ring of hypointensity, an inner ring of intermediate signal intensity on T1 and high signal intensity on T2-weighted images. But bacteriological investigations are required for definite diagnosis.

Our patient had predominant low back pain probably because of the lesion located more towards the midline in the posterior iliac crest. Clinically there was a vague palpable swelling with tenderness in the posterior ilium adjacent to the sacroiliac joint. The routine haematological profile was inconclusive. Plain anteroposterior radiograph of the pelvis showed an osteolytic lesion in the posterior iliac crest. CT showed well-defined lytic lesion with a breach in the outer cortex. Bone scan and MRI were suggestive of infective pathology.

Low virulent organisms are thought to be the usual infective agents. *Staphylococcus aureus* is cultured in 50% cases, including in our case), but in 20% of cases, the culture is negative. Other organisms often reported are *Streptococcus, Escherichia coli, Pseudomonas* and *Salmonella*. Thorough curettage combined with antibiotic therapy (six weeks) gives excellent outcome in these cases^5^. At the end of 18 months, our patient was asymptomatic without any evidence of recurrence.

In conclusion, ruptured Brodie’s abscess of posterior ilium with gluteal syndrome is very unusual which is often missed or misdiagnosed because of its subtle clinical presentation. Strong clinical suspicion along with the knowledge of possible pelvis and sacroiliac joint pathologies will help the treating clinicians while evaluating cases of paediatric low back pain.
